# Simultaneous Screening of Multiple Mutations by Invader Assay Improves Molecular Diagnosis of Hereditary Hearing Loss: A Multicenter Study

**DOI:** 10.1371/journal.pone.0031276

**Published:** 2012-02-24

**Authors:** Shin-ichi Usami, Shin-ya Nishio, Makoto Nagano, Satoko Abe, Toshikazu Yamaguchi, the Deafness Gene Study Consortium

**Affiliations:** 1 Department of Otorhinolaryngology, Shinshu University School of Medicine, Asahi, Matsumoto, Japan; 2 Department of Clinical Genomics, Biomedical Laboratories, Inc., Matoba, Kawagoe-shi, Saitama, Japan; Instituto de Ciencia de Materiales de Madrid - Instituto de Biomedicina de Valencia, Spain

## Abstract

Although etiological studies have shown genetic disorders to be a common cause of congenital/early-onset sensorineural hearing loss, there have been no detailed multicenter studies based on genetic testing. In the present report, 264 Japanese patients with bilateral sensorineural hearing loss from 33 ENT departments nationwide participated. For these patients, we first applied the Invader assay for screening 47 known mutations of 13 known deafness genes, followed by direct sequencing as necessary. A total of 78 (29.5%) subjects had at least one deafness gene mutation. Mutations were more frequently found in the patients with congenital or early-onset hearing loss, i.e., in those with an awareness age of 0–6 years, mutations were significantly higher (41.8%) than in patients with an older age of awareness (16.0%). Among the 13 genes, mutations in *GJB2* and *SLC26A4* were mainly found in congenital or early-onset patients, in contrast with mitochondrial mutations (12S rRNA m.1555A>G, tRNA(Leu(UUR)) m.3243A>G), which were predominantly found in older-onset patients. The present method of simultaneous screening of multiple deafness mutations by Invader assay followed by direct sequencing will enable us to detect deafness mutations in an efficient and practical manner for clinical use.

## Introduction

From a series of etiological studies, 60–70% of childhood hearing loss has been estimated to be of genetic etiology, with the rest due to environmental causes, including newborn delivery trouble, acoustic trauma, ototoxic drug use, and prenatal/postnatal infection [1]. However, until now, there has been no multicenter study based on genetic testing. Along with early discovery of hearing loss by newborn hearing screening programs and subsequent intervention programs, much attention has been paid to the determination of the hearing loss etiology. Therefore, genetic testing has become more important for highly accurate diagnosis, prediction of severity of hearing loss, estimation of associated abnormalities, selection of appropriate habilitation options, prevention of hearing loss, and better genetic counseling. Although more than one hundred loci have been mapped and 46 genes reported to be responsible for hereditary hearing loss (Hereditary Hearing Homepage; http://webh01.ua.ac.be/hhh/), many may cause similar phenotypes without any abnormality other than hearing loss. This genetic heterogeneity has made clinical application difficult, in spite of the considerable advances in discovery of deafness genes. We have previously established a screening strategy focusing on recurrent mutations and demonstrated its benefits for clinical application [2]. We carried out the current multicenter study to determine 1) whether the simultaneous screening of the multiple deafness mutations by Invader assay is applicable for clinical use, 2) whether the genetic etiology is truly prevalent among hearing loss patients and 3) whether genetic causes differ by ages.

## Materials and Methods

### Subjects and clinical status

As summarized in Table 1, two hundred sixty-four Japanese patients with bilateral sensorineural hearing loss from 33 ENT departments nationwide participated in the present study. We first applied the Invader assay for screening forty-seven known mutations of 13 known deafness genes, followed by direct sequencing as necessary.

**Table 1:** Clinical features of subjects in this study.

	*Total (n = 264)*	*Early onset (n = 141)*	*Late onset (n = 100)*
Severity of HL			
normal – moderate	148	58	78
severe – profound	95	70	21
unknown	21	13	1
Inheritance			
AD or Mitochondrial	38	9	24
AR or Sporadic	119	69	42
unknown	107	63	34
Other clinical features			
inner ear malformations	52	37	10
EVA	30	22	4
goiter	8	4	3
diabetes mellitus	14	3	11

HL: Hearing loss.

AD: Autosomal dominant.

AR: Autosomal recessive.

EVA: Enlarged vestibular aqueduct.

Hearing loss was evaluated using pure-tone audiometry (PTA) classified by a pure-tone average over 500, 1000, 2000 and 4000 Hz in the better hearing ears. For children who were unable to be tested by PTA, we used an average over 500, 1000, 2000 Hz in either auditory steady-stem response (ASSR) or conditioned oriented reflex audiometry (COR), or the response threshold (dB) from auditory brainstem response (ABR). Computed tomography (CT) scans were performed to check for congenital inner ear anomalies.

Status of hearing loss in the 264 patients was: mild (21–40 dB) in 39 patients (14.7%), moderate (41–70 dB) in 84 (31.8%), severe (71–94 dB) in 39 (14.8%) and profound (>95 dB) in 56 patients (21.2%). Twenty-four subjects were classified as having normal hearing due to a specific audiogram with hearing loss only in the high or low frequency portions. With regard to onset age (the age of awareness), 141 patients had early onset deafness (below 6 y.o.), 100 had late onset deafness, and the rest had unknown onset ages.

The inheritance composition of the subjects was as follows: 38 subjects from autosomal dominant or mitochondrial inherited families (two or more generations affected); 119 subjects from autosomal recessive families (parents with normal hearing and two or more affected siblings) or subjects with sporadic deafness (also compatible with recessive inheritance or non-genetic hearing loss). None of the patients had an X-linked pattern of inheritance. The numbers of patients with other manifestations were inner ear malformations (52), enlarged vestibular aqueduct (EVA) (30), goiter (8), and diabetes mellitus (14). None of the patients had typical clinical features of Usher syndrome or BOR syndrome.

All subjects gave prior informed consent for participation in the project and the Ethical Committee of Shinshu University as well as the relevant bodies of the participating institutions of the Deafness Gene Study Consortium approved the study.

### Invader assay

Invader technology is convenient for mutation genotyping, offering a simple diagnostic platform to detect single nucleotide changes with high specificity and sensitivity from unamplified genomic DNA.

We applied the Invader assay for screening forty-seven known mutations of 13 known deafness genes [*GJB2*(NM_004004.5), *SLC26A4*(NM_000441.1), *COCH*(NM_001135058.1), *KCNQ4*(NM_172163.2), *MYO7A*(NM_000260.3), *TECTA*(NM_005422.2), *CRYM*(NM_001888.3), *POU3F4*(NM_000307.3), *EYA1*(NM_172060.2), mitochondrial 12 s ribosomal RNA, mitochondrial tRNA(Leu), mitochondrial tRNA(Ser), and mitochondrial tRNA(Lys)] (Table 2). Mutations were selected on the basis of a mutation/gene database established in the Japanese deafness population. The detailed methodological protocol was described elsewhere [2]. In brief, 1.2 ul of primary probe/Invader oligonucleotides mixture (containing 0.5 umol/l wild type primary probes, 0.5 umol/l mutant primary probe, 0.05 umol/l Invader oligonucleotide, and 10 mmol/l MOPS) were poured into each well of 384-well plates. Fluorescent resonance energy transfer (FRET)/Cleavase mixture (Third Wave Technologies, Madison, WI) was added to the probe/Invader oligonucleotide-containing plates. Then, 3 ul of 5–100 fmol/l synthetic target oligonucleotides (positive control), 10 ug/ml yeast tRNA (no target control), and denatured genomic DNA samples (>15 ng/ul) were added. Next, 6 ul of mineral oil (Sigma, St. Louis, MO) were overlayed into all reaction wells and incubated at 63°C for 4 hour. After incubation fluorescence was measured by a Cyto Fluor 4000 fluorescent micro plate reader (Applied Biosystems, Foster CA). The heteroplasmy rate for mitochondrial mutations was quantified by detection of fluorescently labeled and digested PCR products through a fluorescence imaging system [2].

**Table 2:** Mutation list of Invader based genetic screening test.

*Gene*	*Exon*	*Codon location*	*Nucleotide change*	*Frequency of mutant alleles (n = 528)*	*Number of patients with mutations (n = 264)*
*GJB2*	exon 2	p.L79fs	c.235delC	43 (8.1%)	29 (10.9%)
*GJB2*	exon 2	p.V37I	c.109G>A	7 (1.3%)	6 (2.3%)
*GJB2*	exon 2	p.[G45E; Y136X]	c.[134G>A; 408C>A]	10 (1.9%)	10 (3.8%)
*GJB2*	exon 2	p.G59fs	c.176_191del	3 (0.6%)	3 (1.1%)
*GJB2*	exon 2	p.R143W	c.427C>T	4 (0.8%)	4 (1.5%)
*GJB2*	exon 2	p.H100fs	c.299_300del	5 (0.9%)	5 (1.9%)
*GJB2*	exon 2	p.T123N	c.368C>A	4 (0.8%)	4 (1.5%)
*GJB2*	exon 2	p.T86R	c.257C>G	1 (0.2%)	1 (0.4%)
*GJB2*	exon 2	p.F191L	c.570T>C	0	0
*GJB2*	exon 2	p.I71T	c.212T>C	0	0
*GJB2*	exon 2	p.A49V	c.146C>T	0	0
*GJB2*	exon 2	p.G12fs	c.35delG	0	0
*SLC26A4*	exon 19	p.H723R	c.2168A>G	22 (4.1%)	17 (6.4%)
*SLC26A4*	int 7/exon 8	splice site	c.919-2A>G	2 (0.4%)	2 (0.8%)
*SLC26A4*	exon 7	p.T410M	c.1229C>T	4 (0.8%)	3 (1.1%)
*SLC26A4*	exon 7	p.V306fs	c.917insG	0	0
*SLC26A4*	exon 19	p.T721M	c.2162C>T	0	0
*SLC26A4*	exon 8/int 8	splice site	c.1001+1G>A	0	0
*SLC26A4*	exon 9	p.A372V	c.1115C>T	0	0
*SLC26A4*	exon 5	p.M147V	c.439A>G	1 (0.2%)	1 (0.4%)
*SLC26A4*	int 5/exon 6	splice site	c.601-1G>A	0	0
*SLC26A4*	exon 9	p.K369E	c.1105A>G	1 (0.2%)	1 (0.4%)
*SLC26A4*	exon 15	p.S551fs	c.1652insT	1 (0.2%)	1 (0.4%)
*SLC26A4*	exon 15	p.C565Y	c.1693G>A	0	0
*SLC26A4*	exon 17	p.S666F	c.1997C>T	0	0
*SLC26A4*	exon 19	p.E704fs	2111ins GCTGG	1 (0.2%)	1 (0.4%)
*SLC26A4*	exon 4	p.L108fs	c.322delC	0	0
*SLC26A4*	exon 4	p.P123S	c.367C>T	0	0
*SLC26A4*	exon 10	p.N392Y	c.1174A>T	0	0
*SLC26A4*	exon 17	p.S610X	c.1829C>A	0	0
*SLC26A4*	exon 17	p.S657N	c.1970G>A	0	0
*EYA1*	exon 12	p.D396G	c.1187A>G	0	0
*EYA1*	exon 8	p.R264X	c.790C>T	0	0
*EYA1*	exon 7	p.Y193X	c.579C>G	0	0
*COCH*	exon 5	p.A119T	c.441G>A	0	0
*KCNQ4*	exon 5	p.W276S	c.827G>C	0	0
*MYO7A*	exon22	p.A886fs	c.2656_2664del	0	0
*TECTA*	exon 16	p.R1773X	c.5318C>T	0	0
*TECTA*	exon 20	p.R2121H	c.6063G>A	0	0
Mitochondrial 12S rRNA			m.1555A>G	-	5 (1.9%)
Mitochondrial tRNALeu			m.3243A>G	-	6 (2.3%)
Mitochondrial tRNASer			m.7445A>G	-	0
Mitochondrial tRNALys			m.8296 A>G	-	0
*CRYM*	exon 8	p.K314T	c.941 A>C	0	0
*CRYM*	exon 8	p.X315Y	c.945 A>T	0	0

### Direct sequencing

Dominant mutations and mitochondrial mutations are themselves diagnostic criteria for molecular diagnosis. But a hallmark of recessive mutations, in *GJB2* and *SLC26A4* for example, is the detection of two mutations in the paternal and maternal alleles. In this study, direct sequencing was further carried out as follows: 1) *GJB2* mutation analysis for all subjects, because the authors wanted to clarify whether the number of mutations on the invader panel are enough (saturated) or not. 2) *SLC26A4* mutation analysis for all the subjects with EVA, 3) *SLC26A4* mutation analysis for heterozygous patients for these genes. DNA fragments containing the entire coding region were sequenced as described elsewhere [3], [4].

## Results

The mutations found by Invader assay and direct sequencing in this study are summarized in Table 2 and 3.

**Table 3:** Mutation list found by direct sequencing analysis.

*Gene*	*Exon*	*Codon location*	*Nucleotide change*	*Frequency of mutant alleles (n = 528)*	*Number of patients with mutations (n = 264)*
*GJB2*	exon 2	p.T8M	c.23C>G	1 (0.2%)	1 (0.4%)
*GJB2*	exon 2	p.K12fs	c.35insG	1 (0.2%)	1 (0.4%)
*GJB2*	exon 2	p.F106Y	c.317T>A	1 (0.2%)	1 (0.4%)
*GJB2*	exon 2	p.A171fs	c.511insAACG	2 (0.4%)	2 (0.8%)
*GJB2*	exon 2	p.C174S	c.522G>C	1 (0.2%)	1 (0.4%)
*SLC26A4*	exon 14	p.S532I	c.1595G>T	2 (0.4%)	2 (0.8%)
*SLC26A4*	exon 16	p.R581S	c.1743G>C	1 (0.2%)	1 (0.4%)
*SLC26A4*	exon 17	p.V659L	c.1975G>C	2 (0.4%)	2 (0.8%)
*SLC26A4*	exon 10	p.L407fs	c.1219delCT	1 (0.2%)	1 (0.4%)
*SLC26A4*	exon 15/int 15	splice site	c.1931+5 G>A	5 (0.9%)	4 (1.5%)

### Invader Assay

A total of 74 (28.0%) hearing-impaired subjects (n = 264) were found to have at least one deafness gene mutation. Among the deafness genes situated on the present diagnostic panel, mutations were most frequently found in the *GJB2* gene. Screening of *GJB2* showed mutations of one or both alleles of the gene in 43 (43/264; 16.2%) samples from the subjects, of which 13 cases had only a single mutation, and 30 cases were compound heterozygotes or homozygotes, confirmed by segregation analysis (Table 4). The most common mutation was c.235delC, accounting for nearly 67% (29/43) of all *GJB2* mutated patients. On the other hand, the *GJB2*: c.35delG mutation, which is known to be the most common mutation in Caucasian or other ethnic populations, was not found in this group. The second most common group of *GJB2* mutations consisted of p.[G45E; Y136X], p.V37I, and c.299_300del. These mutations were detected in more than 5 patients each, and their allele frequencies were relatively high. Three mutations (p.T86R, p.R143W, and c.176_191del) were observed in more than one patient. p.F191L, p.I71T, p.A49V and c.35delG mutations were not found. One pair of p.[G45E; Y136X] mutations was detected among 10 persons in a heterozygous state. Subsequent parental DNA segregation study through direct sequencing indicated two mutations were in *cis*. The p.T123N mutation was found in 4 subjects but, based on our recent study, is not likely to be a pathologic mutation [5].

**Table 4:** Diagnostic efficiency of Invader assay alone and Invader assay and direct sequencing.

	*Total (n = 264)*	*Early onset (n = 141)*	*Late onset (n = 100)*
Invader assay alone			
*GJB2* homozygote/compound heterozygote	30 (11.4%)	29 (20.6%)	1 (1.0%)
*GJB2* heterozygote	13 (4.9%)	7 (5.0%)	6 (6.0%)
*SLC26A4* homozygote/compound heterozygote	9 (3.4%)	9 (6.4%)	0 (0%)
*SLC26A4* heterozygote	14 (5.3%)	10 (27.1%)	2 (2.0%)
Mitochondria A1555G	5 (1.9%)	2 (1.4%)	2 (2.0%)
Mitochondria A3243G	6 (2.2%)	1 (0.7%)	5 (5.0%)
Total	74 (28.0%)*	55 (39.0%)*	16 (16.0%)
Invader assay and direct sequencing			
*GJB2* homozygote/compound heterozygote	33 (12.5%)	31 (21.9%)	2 (2.0%)
*GJB2* heterozygote	13 (4.9%)	7 (5.0%)	5 (5.0%)
*SLC26A4* homozygote/compound heterozygote	18 (6.8%)	18 (12.7%)	0 (0%)
*SLC26A4* heterozygote	7 (2.7%)	4 (2.8%)	2 (2.0%)
Mitochondria A1555G	5 (1.9%)	2 (1.4%)	2 (2.0%)
Mitochondria A3243G	6 (2.2%)	1 (0.7%)	5 (5.0%)
Total	78 (29.5%)**	59 (41.8%)**	16 (16.0%)

*Three cases carried double mutations (cases 1 to 3 in Table 5).

**Four cases carried double mutations shown in Table 5.

The second most frequent gene with mutations was the *SLC26A4* gene (23/264; 8.7%). Five cases were homozygotes of p.H723R, one was a homozygote of p.T410M, 3 were compound heterozygotes, and 14 had only one mutation of *SLC26A4* (Table 4). Of the 19 *SLC26A4* mutations, 12 (c.917insG, p.T721M, c.1001+1G>A, p.A372V, c.601-1G>A, p.C565Y, p.S666F, c.322delC, p.P123S, p.N392Y, p.S610X, and p.S657N) were not found in any samples, but the remaining 7 *SLC26A4* mutations were confirmed in more than one subject. Especially, the p.H723R mutation was found to be in high allele frequency (4.1%). All of the patients with *SLC26A4* mutations had EVA, which has been demonstrated to be a result of the mutations of this gene. *SLC26A4* mutations were detected by Invader assay in 63.6% of the patients with EVA.

Mitochondrial m.1555A>G mutations were found in 1.9% (5/264) of the patients and the m.3243A>G mutation was identified in 2.3% (6/264).

Mutations in nine deafness genes (*COCH*, *KCNQ4*, *MYO7A*, *TECTA*, *CRYM*, *POU3F4*, *EYA1*, mitochondrial tRNA(Lys) m.8296A>G, mitochondrial tRNA(Ser) m.7445A>G) were not identified in any patients (Table 2).

Notably, 4 subjects were found to have double gene mutations. Two cases were *SLC26A4* compound heterozygous or homozygous mutations with a *GJB2* heterozygous mutation. One case was a compound heterozygous of *GJB2* with a *SLC26A4* heterozygous mutation and the remaining case was a *GJB2* homozygous mutation with a mitochondrial 1555A>G mutation (Table 5).

**Table 5:** Double mutation cases found in simultaneous mutation screening.

*Genotype*	*Patients Number*
*GJB2*:p.[V37I];[V37I]; Mitochondria m.1555A>G	1 (0.4%)
*GJB2*:c.[235delC];p.[R143W]; *SLC26A4*:p.[M147V]	1 (0.4%)
*GJB2*:p.[V37I]; *SLC26A4*:p.[H723R];[ H723R]	1 (0.4%)
*GJB2*:p.[F106Y]; *SLC26A4*:p.[H723R]; c.[1931+5G>A]	1 (0.4%)
Total	4 (1.5%)

**Figure 1:**
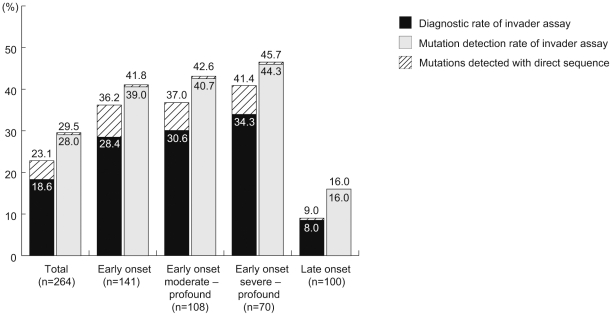
Detection rate by onset/awareness age and severity of hearing loss. Diagnostic rates and detection rates of this simultaneous multiple mutations screening and direct sequencing for biallelic mutations in autosominal recessive genes or mitochondrial mutations increased when restricted to congenital/early-onset hearing loss, and moderate or severe hearing loss. Combined direct sequence and invader screening enhanced the diagnostic rate but not the mutation detection rate.

The detection rate of mutations was 40.4% for the patients with congenital or early-onset hearing loss, i.e. in those with an awareness age of 0∼6 years. The rate in congenital hearing loss patients also increased when restricting to the patients with moderate or more severe hearing loss (>50 dB; 40.7%) or severe hearing loss (>70 dB; 44.3%) (Fig. 1). In contrast, the detection rate was only 16.0% in the patients with an older age of onset/awareness (Fig. 1). Among the 13 included genes, mutations is *GJB2* and *SLC26A4* were mainly found in congenital patients or early-onset patients, in contrast with mitochondrial mutations, such as 12S rRNA m.1555A>G or tRNA(Leu(UUR)) m.3243A>G, which were predominantly found in older-onset patients (Table 4). The p.V37I mutation in the *GJB2* gene was also found in older-onset patients (data not shown).

**Figure 2:**
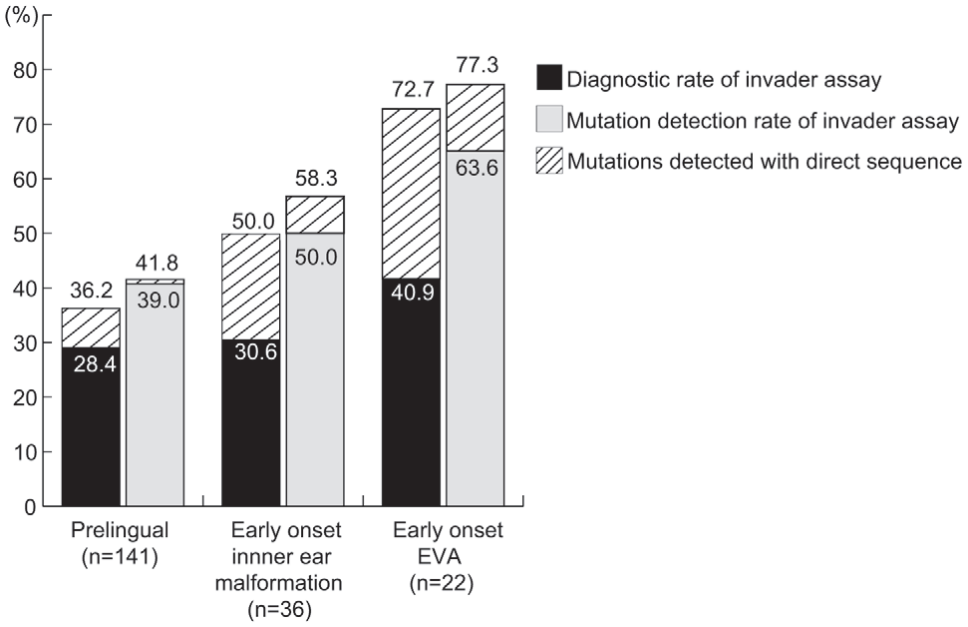
Radiographic findings and detection rate. Detection rate was elevated when subjects were restricted to those with inner ear anomaly or EVA. Combined direct sequence and invader screening enhanced the diagnostic rate but not the mutation detection rate.

With regard to the relationship between radiographic findings and genetic testing, the mutation detection rate was elevated when restricting to the patients with inner ear anomaly (50.0%) and EVA (63.6%) (Fig. 2).

### Direct sequencing

Direct sequencing identified 9 mutations in 15 cases which were not included in the Invader assay panel and improved the mutation detection/diagnostic rate obtained by Invader assay analysis (28.0%/18.6%) to 29.5%/22.7%. (Fig. 1). Combining direct sequencing with invader screening enhanced the diagnostic rate notably but not the mutation detection rate. In detail, direct sequencing identified additional mutations in three cases with single *GJB2* mutations by Invader assay that were finally diagnosed as compound heterozygous mutations of *GJB2* (p.[T86R]; c.[511insAACG], p[T8M];[V37I] and c.[35insG];[235delC]).

In 7 cases only a single *SLC26A4* mutation was found by invader assay, and additional mutations were found by direct sequencing (two cases of p.[H723R];c[1931+5G>A] and one each cases of p.[R581S];[H723R], p.[V659L];[H723R], p.[S532I]; c.[2111insGCTGG], p.[T410M]; c.[1931+5G>A] and p.[K396E];[S532I]). Two cases carried EVA but without any mutations found in Invader assay, c[1931+5G>A]; [1931+5G>A] and p.[V659L];c[1219delCT] compound heterozygous mutations were found by direct sequencing. With the combination of Invader assay and direct sequencing, and restriction to patients with EVA, the mutation detection rate was elevated to 17/22 cases (77.3%, Fig. 2). Fifteen of them carried homozygous or compound heterozygous *SLC26A4* mutations.

## Discussion

We previously reported that simultaneous detection of common deafness gene mutations has excellent sensitivity and accuracy [2]. In this study, using samples from patients at 33 institutions nationwide from northern to southern Japan, we confirmed that the Invader assay based on the Japanese deafness gene mutation database works efficiently in the clinical base to detect the responsible gene mutations from the patients with various onset/awareness ages. We detected mutations in 29.5% overall, and the 41.8% detection rate for congenital or early onset sensorineural hearing loss was especially remarkable. A series of epidemiological studies have demonstrated that genetic disorders are common causes of congenital deafness and it is estimated that 60–70% of the etiology may be caused by genetic factors [1]. Genetic testing is crucial to diagnose the etiology, but more than 100 genes are estimated to be involved and such genetic heterogeneiety has hampered the genetic testing for deafness as a routine clinical test. The present detection rate; i.e., 41.8%, is a strikingly good rate for a clinical application, and it is expected that clinical deafness mutation screening will greatly improve medical management and facilitate extensive genetic counseling for hearing impairment. Additional direct sequencing, as well as a new version of the screening panel which includes novel identified mutations, will likely improve the detection rate. For the older ages of onset, the detection rate was comparatively low (16.0%). Probably this is due to the panel mainly including responsible genes for congenital deafness but not the responsible genes for late onset hearing loss. An alternative explanation may be that environmental factors may be involved in this group of deafness patients.

The present study confirmed that mutations in three genes, *GJB2*, *SLC26A4*, and the mitochondrial 12 s rRNA, are so far the major known causes of hereditary hearing loss nationwide in Japanese [6], and thus much attention should be paid to these genes when performing genetic testing of hearing loss patients.

The most frequently found were mutations in the *GJB2* gene. This gene is so far the most common responsible gene for congenital deafness worldwide [7]. The detection rates (17.4% for all, 27.0% for congenital) are in accordance with our previous data of 15% in the overall deafness population and 25% in congenital deafness patients [5]. The mutation spectrum found in this study is also in accordance with our previous results [2], [4], [5]. In *GJB2* screening, 46 (17.4%) samples from deafness subjects had mutations of one or both alleles of the *GJB2* gene. As expected from the above reports, the c.235delC mutation was found to be the most prevalent mutation in our screening, accounting for 10.9% (29 of 264) of the hearing-impaired persons. Fourteen patients were c.235delC homozygotes and 11 were compound heterozygotes having c.235delC, confirmed by segregation analysis, and 4 patients were c.235delC heterozygotes without a second mutation. Direct sequencing identified novel mutations (p.T8M, c.35insG, p.F106Y, p.C174S and c.512insAACG) in the patients with a single mutation detected by Invader assay (Table 3).

Many benefits of *GJB2* gene genetic testing have been pointed out. There have been general rules that inactivating mutations (deletion mutations and stop mutations) show more severe phenotypes compared to those caused by non-inactivating mutations (missense mutations) [5], [8], [9]. As well as a highly accurate diagnosis, these genotype-phenotype correlation data could provide prognostic information to help decide the strategy of intervention with hearing, i.e., whether a child should receive cochlear implantation or hearing aids. For the patients with severe phenotypes who have *GJB2* mutations, genetic information would aid decision-making regarding cochlear implantation, because their hearing loss is of cochlear origin and they therefore are good candidates for implantation. In fact, cochlear implantation has resulted in remarkable improvement in auditory skills and development of speech production for patients with profound hearing loss associated with *GJB2* mutations [10].

In the *SLC26A4* gene, 7 cases were homozygotes, 11 cases were compound heterozygotes, and 7 cases had only one mutation (Table 4). Of the 19 *SLC26A4* mutations, 12 were not found in any samples, but the remaining 7 mutations were all confirmed in more than one patient. Especially, the p.H723R mutation was found to be in high allele frequency (4.1%). Direct sequencing identified novel mutations (c.1931+5G>A, p.S532I, p.R581S, p.V659L) in the patients with a single mutation by Invader assay and c.1219delCT mutation in a patient with EVA (Table 3).

As in our previous study [2], *SLC26A4* mutations were found only in the patients with EVA, suggesting a phenotype of hearing loss with EVA can be a diagnostic indicator of this category of disease.

Fluctuation and progressiveness of hearing loss are characteristic of hearing loss associated with EVA [11], [12] and the early detection of *SLC26A4* mutations enables prediction of these clinical symptoms. Genetic testing is also useful in estimating associated abnormalities (goiter), selection of appropriate habilitation options, and better genetic counseling. In some cases, goiter is evident during the teen years [12]. In this study, 8 patients had hearing loss and goiter and 4 of them carried homozygous or compound heterozygous *SLC26A4* mutations.

In recessive mutations such as *GJB2* and *SLC26A4*, detection of two mutations in the paternal and maternal alleles is a hallmark. In the present “two step” screening method Invader assay is first performed followed by direct sequencing. As seen in Figs. 1 and 2, most of the mutations were successfully detected by the first Invader screening and the additional direct sequencing improved the “diagnostic” rate. This is very important to find the first mutation for identifying the responsible gene and the results indicate this screening is technically efficient. Difficult cases of a heterozygous state without a second mutation are also seen [4], [5], [13], [14]. As previously reported, in a substantial proportion of patients our Invader techniques and additional direct sequencing revealed only one mutant *GJB2* or *SLC26A4* allele causing deafness by recessive pattern. We believe that there is one more occult mutation somewhere because the frequency of heterozygous patients was much higher than that of mutation frequency in the control population. Another explanation may be the high frequency of carriers in the population. But given the carrier frequency in normal controls, the number of heterozygous deafness cases was greater than would be expected. Second mutations may be present in the same gene or genes in the same chromosomal region. Recent statistical analysis has shown that one allele mutation of *GJB2* and *SLC26A4* is more likely to be a pathological status than a carrier status [15] and indeed, patients with one *SLC26A4* mutation are associated with EVA, therefore it is strongly likely that there is a second mutation within this gene. Another possibility is that mutations in the regulatory region may be involved in phenotypic expression [16].

The m.1555A>G mutation in the mitochondrial 12SrRNA gene, which was found in 5 4 subjects, was mainly found in those with older onset age. This mutation has been reported to be associated with aminoglycoside injection and found in 3% of the patients who visited the outpatient clinic [17], [18]. The current findings are compatible with our previous report that this mutation is a frequently encountered cause for postlingual deafness in patients who received cochlear implantation [18]. This mutation was also found in the congenital or early onset age group as well, in line with our previous study [2]. It is likely that there is a considerably large high-risk population worldwide and a rapid screening method as well as careful counseling should be established to prevent aminoglycoside-induced hearing loss in this group.

The m.3243A>G mutation in the tRNA(Leu (UUR)) gene was found in 6 patients in the older-onset group. This mutation was first reported at a high frequently in the patients with clinical manifestations of MELAS [19], and has also been found in diabetes mellitus patients [20]. It is known to be commonly associated with hearing loss patients (especially with diabetes mellitus) [21]. The hearing loss is adult onset, symmetric high frequency involved [22]. In this study, all 6 patients with this mutation were associated with diabetes mellitus and progressive hearing loss. Five patients had maternally inherited hearing loss (the mother also had hearing loss), but one subject was a sporadic case (the mother did not have hearing loss from the anamnestic evaluation) and therefore is unlikely to be a mitochondrial candidate from clinical evaluation. The present multigene screening is also unexpectedly efficient for such atypical cases.

Heteroplasmy is one of the significant factors determining the expression of mitochondrial disease. The Invader assay is comparatively accurate at detecting the heteroplasmic rate [2], and the present two patients with the 3243 mutation showed 3% and 24% heteroplasmic rates.

In contrast to the three genes discussed above, mutations of the *COCH, KCNQ4, MYO7A, TECTA, CRYM, POU3F4 and EYA1* genes were not found in the present deaf subjects in line with our previous study [2]. This is likely due to them being very rare and usually independent mutations found in only one family. Although analysis for these mutations should be performed to identify the molecular nature of deafness as the first deafness screening step, a different strategy may be necessary for screening for them.

In conclusion, the simultaneous examination of the multiple deafness mutations by Invader assay followed by direct sequencing if necessary, will enable us to detect deafness mutations in an efficient and practical manner for clinical use. This screening strategy will facilitate more precise clinical genetic diagnosis, appropriate genetic counseling and proper medical management for auditory disorders. Against this background, since 2008 the Ministry of Health and Welfare of Japan has allowed this screening to be performed as an advanced medical technology.

A Japanese summary of this article has been provided as Supporting Information (Japanese summary S1).

## Supporting Information

Japanese Summary S1 **Simultaneous Screening of Multiple Mutations by Invader Assay.** The present method of simultaneous screening of multiple deafness mutations by Invader assay followed by direct sequencing will enable us to detect deafness mutations in an efficient and practical manner for clinical use.
(PDF)
