# Supplementary material for: Simultaneous Screening of Multiple Mutations by Invader Assay Improves Molecular Diagnosis of Hereditary Hearing Loss: A Multicenter Study
Source: PLoS One. 2012 Feb 24;7(2):e31276. doi: 10.1371/journal.pone.0031276 (PMC3286470; doi:10.1371/journal.pone.0031276)
Supplement: Japanese Summary S1 — Simultaneous Screening of Multiple Mutations by Invader Assay. The present method of simultaneous screening of multiple deafness mutations by Invader assay followed by direct sequencing will enable us to detect deafness mutations in an efficient and practical manner for clinical use. (PDF) [file pone.0031276.s001.pdf]

インベーダーアッセイを用いた難聴の遺伝子診断：全国多施設共同研究

宇佐美真一[1)]
西尾信哉[1)]
長野真[2)]
阿部聡子[2)]
山口敏和[2)]
難聴遺伝子研究コンソーシアム[3*)]

１：信州大学医学部耳鼻咽喉科
２：(株) ビー・エム・エル 総合研究所　先端技術開発本部
３：難聴遺伝子研究コンソーシアム
（北海道大学、弘前大学、岩手医大、盛岡市立病院、東北大学、山形大学、福島医大、自治医大、群馬大学、虎の門病院、あべ耳鼻科、慈恵医大、日本医大、日本医大多摩永山病院、日本大学、北里大学、浜松日赤、三重大学、和歌山医大、滋賀県立小児保健医療センター、大阪医大、兵庫医大、神戸市民病院、岡山大学、山口大学、愛媛大学、福岡大学、九州大学、長崎大学、神田耳鼻科、宮崎大学、鹿児島大学、琉球大学、ビー・エム・エル（株）研究開発本部、信州大学医学部社会予防医学講座）

はじめに

近年のヒトゲノム解析研究の発展により、多くの原因遺伝子が同定され報告されるようになり、もはや難聴は原因不明の疾患ではなくなってきている。疫学的な研究により従来から先天性難聴の 60-70％は遺伝子の関与によるものと推測されているが、難聴の原因を知るためには遺伝子診断が必要不可欠になってきている。難聴は多種類の遺伝子が「難聴」という同じ表現型をとる（遺伝子異質性：locus heterogeneity）ために、難聴を主訴に外来を受診した患者がどの原因遺伝子が関与しているかを推測することは困難である。原因遺伝子の数に関しては従来から数十から 100 ほどの原因遺伝子が推測されており、多数の変異を同時に効率的に検索する必要がある。インベーダー法は日本人に特徴的なあるいは頻度の多い遺伝子変異を網羅的にスクリーニングできるスクリーニング法として注目されている。今回の研究では全国 33 施設の協力のもとにインベ

ーダー法によるスクリーニングを行い、全国規模での疫学的研究を行うとともに、難聴の遺伝子診断の臨床応用に関してその有用性を検証した。

対象と方法

全国33施設から集められた日本人の両側感音難聴患者264例について、13遺伝子47変異についてインベーダー法を用い検索した。またインベーダー法で変異の見出された症例に関しては直接シークエンス法にてさらに変異検索を行った。

患者の内訳を表１に示すが、発症年齢(難聴に気づいた年齢)は先天性・小児期発症(141例)、後天発症(100例)、不明(23例)であった。難聴の程度については、軽度(21-40dB) 14.7%、中等度（41-70dB）31.8%、高度（71-94dB）14.8%、重度（>95dB）21.2%であった。また遺伝形式に関しては常染色体優性遺伝形式／ミトコンドリア遺伝形式が38例、常染色体劣性遺伝形式／孤発症例が119例であった。また随伴症候として内耳奇形52例（前庭水管拡大30例を含む)、糖尿病14例であった。

結果および考察

インベーダー法を用い１次スクリーニングを実施、さらに必要に応じ直接シークエンス法を用いた２次スクリーニングを行い変異確認、新規変異検索を行った結果、難聴患者264名のうち78名（29.5%）に変異が見出され（表２）、多施設共同研究として行っても同等の検出率が得られたことからインベーダー法によるスクリーニングが臨床検査として有用であることが確認された。今回の解析症例で発症年齢が６歳以下の先天性難聴患者に限ると検出率は41.8%（59/141）に上昇した（表４）。また高度難聴に限ると検出率は 45.7%（32/70）とさらに高まり原因検索のための検査としての有用性が確認された（図１）。また内耳奇形を伴う難聴に限ると 58.3%（21/36）、さらに前庭水管拡大症例に限ると77.3%（17/22）と検出率が高まり（図２）、耳鼻咽喉科医が聴覚検査や画像検査と組み合わせて行うことにより診断率が高まることが明らかになった。

「先天性難聴の遺伝子診断」は2008年7月1日付で厚生労働省より先進医療として承認され、現在臨床応用が開始されている。今後、国内で難聴の遺伝子診断が臨床診療として定着し難聴患者の診断、治療に利用されることが期待される。
